# Current and Emerging Strategies for the Prevention of Respiratory Syncytial Virus (RSV) Infections: A Comprehensive Review of Vaccines and Antibody Therapies

**DOI:** 10.7759/cureus.101340

**Published:** 2026-01-12

**Authors:** Diana Genis, Wajhat Riaz, Azhar Hussain, Tamara Oz

**Affiliations:** 1 Pharmaceutical and Biomedical Sciences, Touro University College of Pharmacy, New York, USA; 2 Pediatrics, Touro University College of Pharmacy, New York, USA

**Keywords:** immunocompromised patients, lower respiratory tract infection (lrti), monoclonal antibodies (mabs), mrna vaccine, patho-physiology, prefusion f protein, prevention strategies, respiratory syncytial virus (rsv), rsv vaccination, vector vaccine

## Abstract

Respiratory syncytial virus (RSV) exerts a profound influence on public health worldwide, particularly affecting vulnerable groups such as infants, the elderly, and those with compromised immune systems. Furthermore, RSV presents notable challenges in vaccine development and distribution, underscoring the importance of ensuring equitable access to preventative solutions. The virus's capacity to evade immune defenses complicates both treatment and prevention, emphasizing the critical need for continued research and the implementation of effective vaccination programs. RSV vaccines, such as Arexvy, Abrysvo, and mRESVIA, as well as monoclonal antibodies, such as nirsevimab and clesrovimab, have been developed using platforms like mRNA technology, protein subunits, and viral vectors, each with unique mechanisms of action, immunogenic properties, and safety profiles. Clinical research demonstrates that mRNA-based vaccines, protein-based vaccines, viral vector vaccines, and monoclonal antibodies effectively reduce hospital admissions and the risk of lower respiratory tract disease from RSV.

However, RSV vaccine efficacy varies across age groups due to differences in immune system development and response. RSV vaccine implementation requires rigorous safety monitoring due to concerns over vaccine-associated enhanced respiratory disease (VAERD) and rare adverse effects, such as Guillain-Barré Syndrome (GBS). Comprehensive post-market surveillance and ongoing pharmacovigilance are vital to refining vaccine strategies, ensuring safety for high-risk groups like infants, the immunocompromised, and the elderly, while confirming that the benefits outweigh potential risks. Healthcare providers play a crucial role in advocating for RSV vaccination, and the development of universal RSV vaccines has become a research priority, aiming to deliver broad protection across various populations and strains. As technological advancements continue, the future of preventative measures, including vaccines and monoclonal antibodies, is poised to bring transformative solutions, ultimately reducing the burden of RSV and improving public health worldwide.

## Introduction and background

Respiratory syncytial virus (RSV) is a ubiquitous respiratory pathogen that is the leading cause of both upper respiratory tract infections (URTIs) and lower respiratory tract infections (LRTIs). The symptomatic presentation of RSV depends on the type of infection it causes. URTIs caused by RSV lead to symptoms such as a stuffy nose and an itchy throat. In contrast, LRTIs can lead to more severe presentations, such as bronchiolitis, chronic obstructive pulmonary disease (COPD), and asthma exacerbations. RSV primarily affects infants, young children, the elderly, and individuals with compromised immune systems [1,2]. Part of the *Paramyxoviridae *family, RSV is an enveloped, non-segmented negative-sense RNA virus responsible for significant morbidity and mortality among high-risk groups mentioned earlier [1]. According to the Centers for Disease Control and Prevention (CDC), RSV is responsible for approximately 2.1 million outpatient visits and 58-80,000 hospitalizations among children under the age of 5, and 100-150,000 hospitalizations among adults 60 years of age or older [3].

For decades, effective preventative measures for RSV have remained a significant global health challenge. Vaccines play a crucial role in preventing RSV-related respiratory infections, minimizing hospitalizations, and lightening the financial strain on healthcare systems [4]. The push to develop an RSV vaccine has grown stronger, particularly in light of the rapid advances in mRNA vaccines for COVID-19, highlighting the potential for swift vaccine development and distribution [4,5].

The development of RSV vaccines is of extreme interest for RSV prevention amongst three particular groups: the pediatric, obstetric, and elderly populations [6]. These target groups are also amongst the high-risk populations for infection by RSV and thus, are at the highest risk of developing RSV-associated complications. The importance of RSV vaccines in public health cannot be understated, as they may aid in preventing RSV-associated morbidity and mortality in a significant number of patients within these populations. While the CDC provides an annual estimate of RSV cases in the United States, the actual number of RSV-associated infections is much higher. Due to the common-cold-like presentation of RSV, a large number of individuals may self-medicate and use household remedies as opposed to seeking medical care. Many individuals may not seek physician care until the later stages of the infection, when the symptomatic presentation may become harder to manage. This review offers a detailed overview of RSV vaccines that have received approval and those showing promise, including their mechanisms of action, efficacy, safety profiles, and implications for global public health.

Structure of RSV

As mentioned above, RSV is an enveloped, non-segmented negative-sense RNA virus classified in the family of virus *Paramyxoviridae *and the genus *Pneumovirus*. RSV is split into two major antigenic groups, A and B, which can be further subdivided into 13 genotypes for RSV-A and 20 genotypes for RSV-B. Both subgroups A and B contribute equally and exhibit similar disease severity globally. However, there is substantial debate about differences in clinical severity across RSV subgroups. These differences could be due to variations in structural proteins, with deviations in the G protein domains. The G protein is responsible for RSV attachment to the host cell. The fusion protein (F) is a type I glycoprotein that enables RSV to invade host cells and is synthesized as an inactive, single-chain polypeptide that assembles into a trimer. It becomes active after cleavage by host proteases into a fusion peptide, where it subsequently undergoes a number of structural rearrangements that lead to its insertion into the target cell membrane. RSV-neutralizing antibodies target either G proteins or F glycoproteins. Many of these antibodies are cross-reactive with both subgroups A and B; however, some have shown neutralization activity against RSV A but no or low neutralization activity against RSV B. This may indicate the presence of immunity specific to each subgroup [7,8].

**Figure 1:**
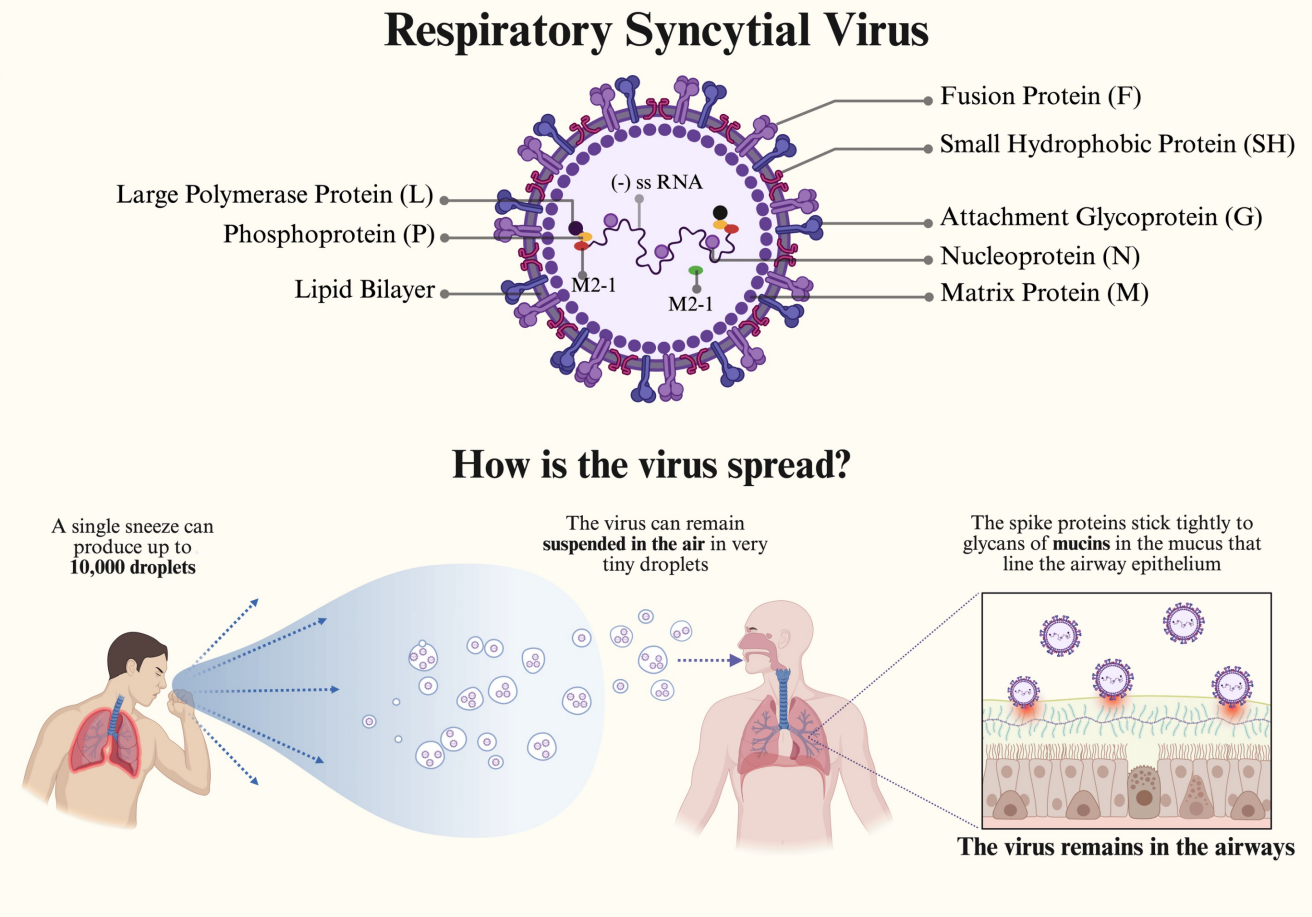
Structure of respiratory syncytial virus (RSV) and transmission Image credits: Azhar Hussain, Diana Genis, Wajhat Riaz, Tamara Oz

The RSV genome comprises 10 genes that encode 11 proteins. Of the 11 proteins, several are extremely important in the transmission of the virus. These include: the matrix protein (M), the small hydrophobic protein (SH), the fusion protein (F), and the attachment protein (G), within the virion (the nucleoprotein (N), phosphoprotein (P), and RNA-dependent RNA polymerase (L)), and two nonstructural proteins (NS1 and NS2). The matrix protein aids in the assembly and stability of RSV virions, while NS1 and NS2 suppress innate immune signaling and counteract apoptotic pathways [9]. These nonstructural proteins help RSV evade the immune system by regulating the host response, including inhibition of the type I interferon response, inhibition of dendritic cell maturation, and induction of an inflammatory state. Furthermore, in respiratory epithelial cells, the fusion protein (F) also helps evade the immune system by inhibiting interferon-λ production induced by interferon regulatory factor 1. In the antiviral immune response to RSV, this is the most vital type III interferon (IFN), and by inducing epidermal growth factor receptor (EGFR) activation, it leads to a continual increase in viral load over time (Figure 1).

The pathogenesis of RSV involves transmission from person to person, mainly through respiratory droplets or fomites. Virions invade the cells that line the airways in the upper respiratory tract via nasopharyngeal or conjunctival mucosa. From the URT, RSV propagates to the LRT, infecting ciliated human airway epithelial cells (hAECs). Subsequently, the virions multiply, leading to these cells detaching and blocking bronchioles in the lower respiratory tract [10]. These obstructions in the bronchioles lead to significant neutrophil infiltration, edema, and inflammation, which further exacerbate airway blockage in the respiratory tract. In infants, the primary manifestation is bronchiolitis, with symptoms consistent with a cold that gradually progress to a persistent cough, increased respiratory rate, and difficulty breathing, and are accompanied by crackles and wheezing on auscultation. In addition to cough, RSV may cause a range of symptoms, such as fever, runny nose, chest tightness, and dyspnea. Furthermore, RSV-associated complications, such as pneumonia, ICU admission, mechanical ventilation, cardiorespiratory complications, and, in some cases, death, are of great concern. Symptoms typically begin three to seven days after infection, with a decrease in mitochondrial respiratory function and an increase in mitochondrial reactive oxygen species (ROS) seen over time. These changes, coupled with RSV’s ability to stabilize hypoxia-inducible factor-1α (HIF-1α) in infected cells, enhance the replication ability and titer of RSV [11,12]. As new virions are formed by genome assembly with viral proteins in the cytoplasm of infected host cells, the F protein mediates the formation of syncytia with neighboring cells, ultimately compromising the integrity of the airway [10].

**Figure 2:**
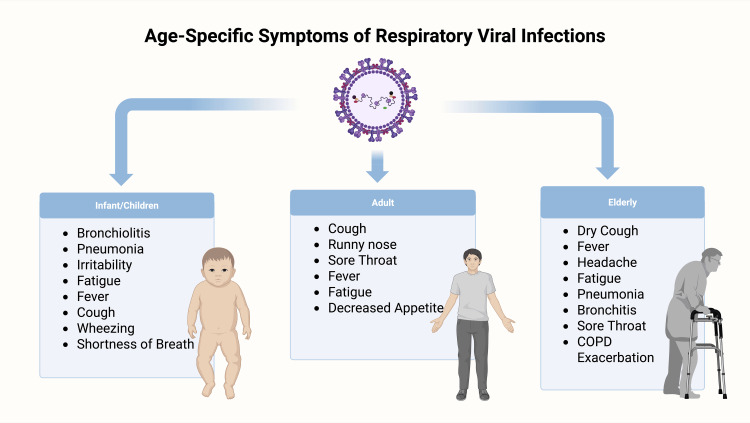
Clinical manifestations across age groups COPD: chronic obstructive pulmonary disease Image credits: Azhar Hussain, Diana Genis, Wajhat Riaz, Tamara Oz

The top five countries with the highest RSV-associated lower respiratory tract infections (India, China, Nigeria, Pakistan, and Indonesia) account for nearly half of the global burden. RSV exhibits seasonality due to geographic variability and meteorological factors, which is important for the development of immunization strategies. In intermediate climate regions, RSV is most prevalent from late autumn to early spring. At the same time, in subtropical and tropical areas, RSV typically circulates for longer, with the highest prevalence during the rainy seasons. Preterm infants, the elderly, and individuals with preexisting conditions (such as cardiac, pulmonary, neurologic, and/or immunological conditions) present with increased rates of illness and death. On a global scale, around 33 million cases of lower respiratory tract infections and 3 million hospitalizations are estimated to be caused by RSV per year [11]. The most severe disease has been observed in infants less than one year of age, especially in low- and middle-income countries, and it has been estimated to cause between 55,000 and 200,000 deaths in children under five years of age annually [10]. In 2019, an estimated 3.6 million RSV-associated lower respiratory infection hospital admissions, 26,300 RSV-associated in-hospital deaths, and approximately 101,000 overall deaths in children under five years globally were reported [13]. Notably, the prevalence of RSV infections and other respiratory infections dropped during the COVID-19 pandemic, possibly due to improved hygiene practices and social distancing [10]. Although public health measures led to an 82% drop in RSV hospitalizations compared to pre-pandemic levels, the prevalence of RSV increased significantly in the aftermath of the pandemic [14]. However, following the reduction of public health restrictions in 2021, RSV exhibited an atypically early and intense resurgence, with hospitalizations among children under five rising to 94,347 during the 2022-2023 season, more than twice the pre-pandemic average [14]. Additionally, children with prior COVID-19 infection had a 40% higher risk of RSV-related medical visits, suggesting a possible impact of SARS-CoV-2 on immune vulnerability [15]. In adults, the RSV disease burden is underestimated due to several factors, including a delay of up to a week in seeking medical care, compared to children, who typically obtain care within 2 to 4 days. Compared with pediatric patients, adults typically exhibit a more gradual onset of clinical symptoms and show reduced sensitivity to diagnostic testing for RSV (Figure 2). Globally, there were an estimated 336,000 RSV-related hospitalizations in older adults in 2019, which is likely underestimated due to previously stated factors. The mortality burden was also found to be significant, with 6.1% of hospitalized older adults 65 years and older resulting in death. Critically, there are few to no global or regional studies that have estimated the burden of RSV in younger adults; thus, increased surveillance and studies across all populations are imperative to truly understand the epidemiology of RSV and the population groups that would benefit most from RSV vaccine administration [16].

RSV treatment is categorized into three main areas: supportive care, immune prophylaxis, and antiviral medications. Most cases of RSV and bronchiolitis do not necessitate specific medical treatment, as numerous historical approaches have proven ineffective. The development of RSV vaccines, monoclonal antibodies, and therapeutic options remains a significant focus of scientific research. The cornerstone of RSV treatment is supportive care, which includes nasal suction and lubrication to ease nasal congestion, antipyretics to manage fever, and assisted hydration when dehydration occurs. Hydration support may be provided orally, via nasogastric tube, or intravenously. Oxygen therapy is utilized for patients experiencing hypoxia. For severe cases with respiratory distress or failure, ventilatory support may be necessary, such as high-flow nasal cannula, continuous positive airway pressure (CPAP), intubation, or mechanical ventilation. Hospitalization is advised for patients with moderate-to-severe symptoms, those requiring supplemental fluids, or those requiring respiratory support.

Palivizumab, a humanized murine monoclonal antibody that targets the RSV membrane fusion protein, may be administered monthly throughout the RSV season and provides passive immune prophylaxis. However, palivizumab is costly; its cost-effectiveness remains controversial, therefore it is scheduled to be discontinued as of December 31, 2025 [17]. Previously, it was only recommended as prophylaxis for children in the first year of life with prematurity less than or equal to 29 weeks' gestational age, chronic lung disease of prematurity, congenital heart disease, or neuromuscular disorders. Ribavirin, a nucleoside analog, is the only antiviral approved for the treatment of RSV in the United States. The use of ribavirin in RSV treatment is controversial, primarily due to its cost, potential risks to healthcare workers exposed during administration, and uncertainties regarding its effectiveness in reducing mortality, the duration of mechanical ventilation required, and the length of hospitalization. The most common adverse reactions in adults were fatigue/asthenia, pyrexia, myalgia, and headache, which were similar to those seen in pediatric patients. Some serious side effects that have been reported include hemolytic anemia, risk of hepatic failure and death, severe hypersensitivity reactions, severe depression and suicidal ideation, suppression of bone marrow function, pancreatitis, and diabetes [18]. While routine use of ribavirin is not recommended, it may be considered selectively based on individual patient circumstances. Ziresovir, a promising antiviral currently in clinical studies, is a potent, selective inhibitor of RSV F protein. Results of a phase 3 trial showed treatment with ziresovir reduced signs and symptoms of bronchiolitis in infants and young children hospitalized with RSV infection, and no safety concerns were identified [19]. Furthermore, various treatment approaches for bronchiolitis have been explored historically, but none have demonstrated consistent, widespread effectiveness in improving clinical outcomes in RSV bronchiolitis. These methods include albuterol, racemic epinephrine, steroids, hypertonic saline, antibiotics, and chest physical therapy. As such, the routine use of these interventions is not advised [2].

## Review

Monoclonal antibodies

Two monoclonal antibodies (mAbs) are of primary clinical interest at this time: nirsevimab and clesrovimab (Table 1). Both target the F protein, but their specificities for different epitopes, pharmacokinetics, and target populations vary. Nirsevimab is a recombinant, long-acting mAb against RSV developed by Sanofi (Paris, France) and AstraZeneca (Cambridge, United Kingdom), with an extended serum half-life and ability to bind a conserved epitope in the prefusion conformation of the F protein [20]. It was approved in 2023 for infants eight months or younger entering their first RSV season and children eight through 19 months of age who remain vulnerable to severe RSV through their second RSV season. This monoclonal antibody is administered as a single intramuscular injection of either 50 mg or 100 mg for neonates and infants entering their first RSV season, depending on the newborn’s weight (see Table 1). For children aged eight through 19 months who remain at risk for severe RSV during their second season, a single 200 mg dose is given as two intramuscular injections [21]. In the Phase III MELODY trial, a single intramuscular dose of nirsevimab reduced the incidence of medically attended RSV-LRTI by 74.5% (95% CI: 49.6-87.1: P:0.001) in healthy late preterm and preterm infants and demonstrated a non-significant 62.1% reduction in RSV-LRTI hospitalization (95% CI: 8.6-86.8: P=0.07) over a 150 day follow-up period [20]. These data, along with results from preterm infant studies, underscore the American Academy of Pediatrics' recommendation for nirsevimab as one of the first-line options for all infants younger than eight months entering their first RSV season and for certain high-risk children up to 24 months of age [22]. Adverse events in this trial and in early post-marketing data have been rare and mostly limited to mild local injection site reactions [23].

**Table 1:** Summary of RSV monoclonal antibodies IM: intramuscular; LRTI: lower respiratory tract infection; MALRI: medically attended lower respiratory tract infection; RSV: respiratory syncytial virus

Monoclonal antibody	Components	Dose	Age group	Efficacy	Safety
Beyfortus^®^ (nirsevimab) [20]	Monoclonal antibody targeting the F protein	50 mg IM for infants < 5 kg; 100 mg IM for infants ≥ 5 kg; 200 mg IM for children 8 - 19 months: Single injection in the anterolateral aspect of the thigh	Infants < 8 months old entering their first RSV season, and children 8-19 months old who remain high-risk entering their first RSV season	Reduction in the risk of MALRI: 79%	Common side effects: rash, pyrexia
Synagis^®^ (palivizumab) [17]	Monoclonal antibody targeting the F protein	15 mg/kg IM in the anterolateral aspect of the thigh every 28-30 days during the RSV season (monthly injections)	Scheduled to be discontinued as of December 31, 2025	Reduction in the risk of RSV hospital admissions: 38 to 86%	
Enflonsia^®^ (clesrovimab-cfor) [24]	Monoclonal antibody targeting the F protein	105 mg IM administered as a single injection	All neonates and infants entering their first RSV season (if born during RSV season). If born outside of season, dose given before the first RSV season	Reduction of MALRI (requiring ≥ 1 indicator of LRTI or severity): 60.5% efficacy in lowering the number of hospitalizations compared to placebo: 84.3%	Common side effects: rash, injection-site erythema + swelling

Enflonsia (clesrovimab-cfor), which was FDA approved in June of 2025, is Merck’s (New Jersey, USA) extended half-life (~44 days) monoclonal antibody with broad activity against RSV-A and RSV-B, targeting the RSV F protein (site IV) to prevent viral fusion and entry into host cells. It is indicated for passive immunization for the prevention of LRTIs from RSV in newborns and infants who are born during or entering their first RSV season only. It uses non-weight-based dosing and represents the first and only RSV preventive measure administered to infants using the same dose regardless of weight. It was formulated to deliver immediate, long-lasting protection for up to five months (the duration of a typical RSV season) using a consistent 105 mg dose across all weight ranges. According to Dr. Ramilo, chair of the Department of Infectious Diseases at St. Jude Children’s Research Hospital, “Enflonsia combines dosing convenience with strong clinical data showing significant reductions in RSV disease incidence and hospitalizations” [24]. It was approved based on results from the Phase 2b/3 double-blind, randomized, placebo-controlled CLEVER trial, which evaluated a single dose of clesrovimab in preterm and full-term infants (birth to one year of age) [24]. It showed that Enflonsia reduced the incidence of RSV-related LRTIs by 60.5% compared to placebo over a five-month period, the study’s primary endpoint (95% CI: 44.2-72.0; p<0.001). Furthermore, Enflonsia reduced RSV-related hospitalizations over a five-month period, a key secondary endpoint, by 84.3% compared to placebo (95% CI: 66.7-92.6; p<0.001), highlighting greater efficacy with increasing disease severity [25].

Safety profiles for both mAbs have been favorable and well characterized. Common adverse effects include fever, rash, and injection-site reactions. These are typically mild and of short duration [26]. Nirsevimab has demonstrated similarly low rates of adverse events in clinical trials and early post-marketing surveillance. Adverse events were limited to rash (0.9%) and injection site reactions (0.3%) [27]. Clesrovimab demonstrated a favorable safety profile in the pivotal CLEVER Phase 2b/3 trial, with most adverse reactions, such as injection site erythema (3.8%), swelling (2.7%), and rash (2.3%), reported as mild or moderate and similar to placebo levels (3.3%, 2.6%, and 1.8%, respectively) [24]. In the SMART Phase 3 trial, safety outcomes in high-risk populations were comparable to those of palivizumab, affirming consistency across diverse infant groups [24]. In all cases, continued pharmacovigilance programs will be crucial to monitoring for any rare or long-term effects, particularly with recently approved drugs.

RSV vaccine types

The development of RSV vaccines has leveraged diverse platforms, each employing unique mechanisms to stimulate immunity. The effective stimulation of immunity is a critical objective in RSV vaccine development, given several complex, interrelated challenges. The virus uses sophisticated methods to bypass innate immune defenses, which delay the body’s initial response mechanisms. Natural RSV infection elicits an adaptive immune response that usually wanes and cannot protect against subsequent infections. Furthermore, the legacy of vaccine-enhanced disease, the absence of an animal model that fully replicates human RSV pathophysiology, and ongoing safety concerns have collectively impeded progress. Addressing these immunological and biological barriers is imperative to accelerate the development of safe and efficacious RSV vaccines [26,28-30]. To overcome these challenges, vaccine development has focused on several advanced platforms, including mRNA, protein subunit, live-attenuated, and viral vector-based approaches (Table 2). Each platform is characterized by unique immunological properties, mechanisms of action, and safety profiles, which confer distinct advantages and limitations. These differences enable targeted applications across diverse populations, such as neonates, older adults, and immunocompromised individuals, while addressing specific healthcare needs and epidemiological considerations [4,28,31,32].

**Table 2:** Summary of RSV vaccines IM: intramuscular; LRTI: lower respiratory tract infection; LRTD: lower respiratory tract disease; RSV: respiratory syncytial virus; PreF3: pre-fusion protein 3; MALRI: medically attended lower respiratory tract infection

Vaccine	Components	Dose	Age group	Efficacy	Safety
Arexvy^®^ [30,31]	Antigen: 120 μg PreF3 and Adjuvant: AS01_E_	0.5 mL IM (deltoid)	Age ≥ 60; AND age 50-59 at increased risk of LRTI	MALRI prevention (57.9-89.0) for season 1 and interim season 2: 77.5%	Common side effects: injection-site pain, fatigue, muscle pain, headache
Abrysvo^®^ [16, 30]	Antigen: 60 μg PreF A OR 60 μg PreF B	0.5 mL IM (deltoid)	Age ≥ 60; AND age 18-59 at increasing risk for LRTD; AND pregnant at 32-36 weeks; AND infants birth to 6 months of age at risk of severe LRTI	MALRI prevention (43-95.2) for season 1 and interim season 2: 81.0%	Common side effects: injection-site pain, fatigue, headache
mRESVIA^®^ [29, 33]	50 µg mRNA encoding PreF protein (subtype A)	0.5 mL IM (deltoid)	Age ≥ 60; AND age 18-59 if increased risk for LRTI	LRTI prevention with 2 or more signs or symptoms: 78.7%; LRTI prevention with 3 or more signs or symptoms: 80.9%	

**Figure 3:**
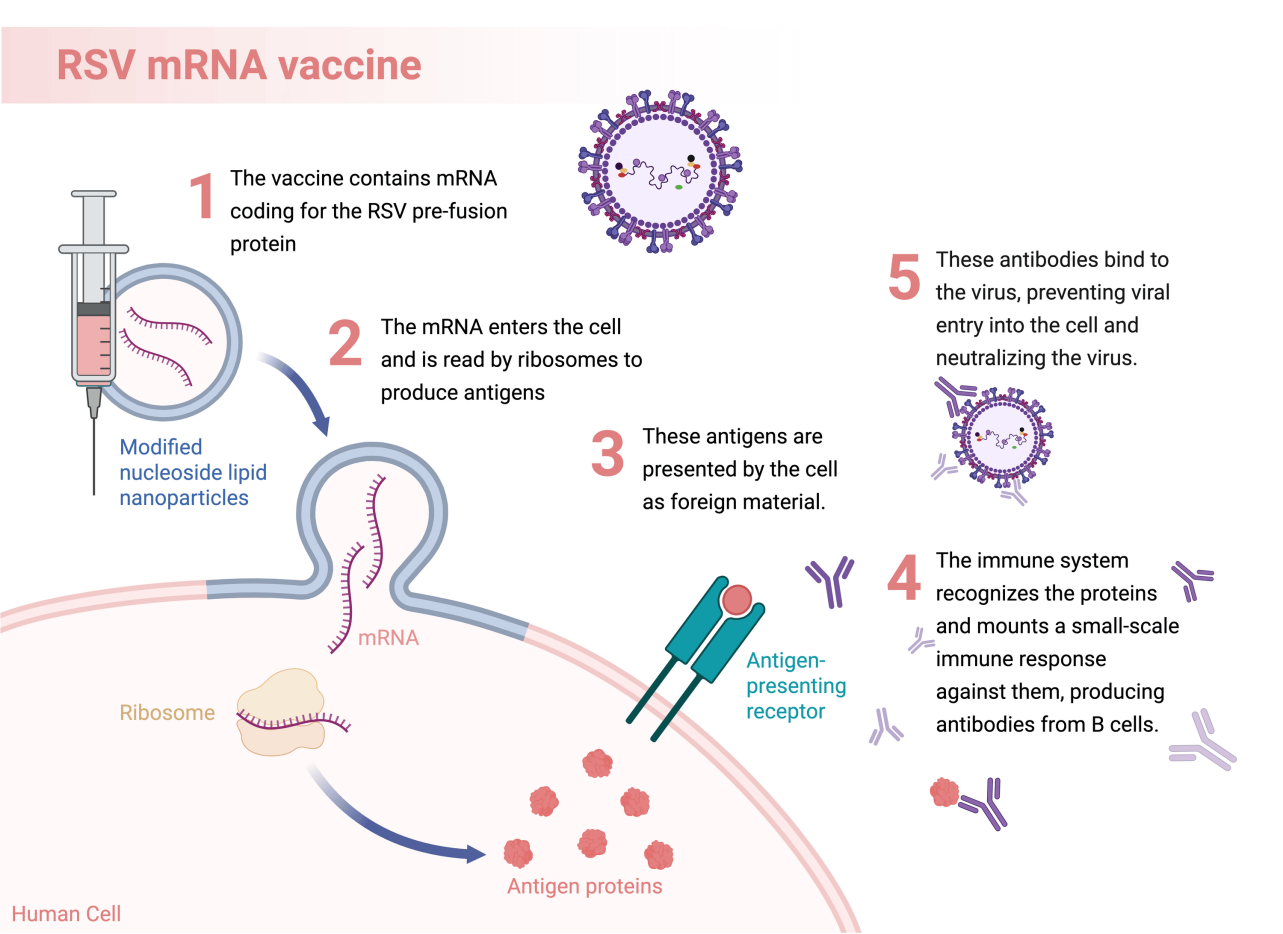
Mechanism of action of RSV mRNA vaccine RSV: respiratory syncytial virus Image credits: Azhar Hussain, Diana Genis, Wajhat Riaz, Tamara Oz

Among these platforms, mRNA vaccines have emerged as a promising strategy for preventing RSV due to their rapid development capabilities and potent immunogenic properties. The mechanism of mRNA vaccines involves the introduction of synthetic messenger RNA that encodes the RSV F protein into host cells (Figure 3). Ribosomes within the cell convert the mRNA into the viral F protein that subsequently appears on the cell surface. The vaccine triggers simultaneous humoral and cellular responses, resulting in neutralizing antibody production and CD4+ and CD8+ T-cell activation, which facilitate the elimination of infected cells and the development of long-term immunity [29]. The successful development of mRNA vaccines for COVID-19 has facilitated rapid progress in RSV mRNA vaccine research, resulting in Moderna’s (Massachusetts, USA) mRESVIA, which elicited strong neutralizing antibody responses and T-cell activation in clinical trials [29]. However, the requirement for ultra-cold storage presents a significant logistical hurdle.

The protein subunit vaccines against RSV contain recombinant prefusion F proteins, which enable host cell entry and stimulate immune protection. They work by presenting viral antigens directly to the immune system, which triggers B-cell activation and antibody production without requiring translation within host cells. Protein subunit vaccines require adjuvants to boost immunogenicity because they elicit limited cell-mediated immunity. Abrysvo, developed by Pfizer (New York, USA), and Arexvy, developed by GlaxoSmithKline (GSK; London, United Kingdom), stand out as leading options because they showed high effectiveness in preventing RSV-related lower respiratory tract disease (RSV-LRTD) in elderly patients [30]. Viral vector vaccines use non-replicating viral vectors, such as adenoviruses, to deliver RSV antigens to host cells, thereby triggering robust cellular immunity and CD8+ T-cell responses. Designed to generate persistent immune memory, these vaccines provide vital protection for vulnerable groups such as older adults and people with weakened immune systems [31].

Adenoviral vector-based vaccines, such as Ad19a, which expresses RSV-F, provide an effective means of generating robust immune responses against the respiratory syncytial virus. The vaccine approach introduces RSV antigens into host cells to generate an immune response that simulates an actual viral infection. The mechanism successfully elicits strong CD8+ T-cell responses and generates neutralizing antibodies, thereby establishing protective immunity [31]. The platform's ability to form durable immune memory is a significant benefit, especially for vulnerable groups such as older people and those with weakened immune systems. The presence of pre-existing immunity to adenoviral vectors can diminish vaccine effectiveness in certain people, posing a primary obstacle to broad use [32].

Different platforms used for RSV vaccines show varying results for both efficacy and safety profiles. Although every RSV vaccine type offers substantial protection against related diseases, they also differ in their ability to induce immune responses, as well as in their adverse event profiles and long-term immunity. mRNA vaccines, such as Moderna’s mRESVIA, elicit strong immune responses by generating neutralizing antibodies and activating CD8+ T cells. Clinical trials confirmed that Moderna’s mRESVIA mRNA vaccination delivers significant protection against lower respiratory tract disease from RSV in high-risk adult populations through a 67.2% efficacy rate for RSV-LRTD and a 78.8% efficacy rate for severe RSV-LRTD [33]. Another study demonstrated an 83.7% success rate in preventing RSV-related lower respiratory tract illness and produced robust neutralizing antibodies and T-cell activation during clinical trials [29]. mRNA vaccines can be quickly adapted for emerging strains, but they face difficulties due to their ultra-cold storage requirements and a tendency to cause adverse reactions, such as mild-to-moderate side effects, which include injection site pain, headaches, and fatigue [34], and they may also exhibit a higher reactogenicity than protein-based vaccines in certain recipients.

Older adults are the ideal candidates for protein-based vaccines due to their durable protection and consistent safety record. These vaccines elicit humoral responses by targeting B-cells to produce neutralizing antibodies. This class of vaccines, exemplified by Pfizer’s Abrysvo and GSK’s Arexvy, showed high efficacy in trials with older adults. Arexvy demonstrated 82.6% efficacy against RSV-LRTD and 94.1% efficacy in preventing severe cases [33]. Similarly, Abrysvo achieved 85.7% efficacy throughout one RSV season [16]. While the safety profiles of these vaccines are well known, the need for adjuvants to improve their immunogenicity does increase the risk of mild local or systemic reactions [30].

**Figure 4:**
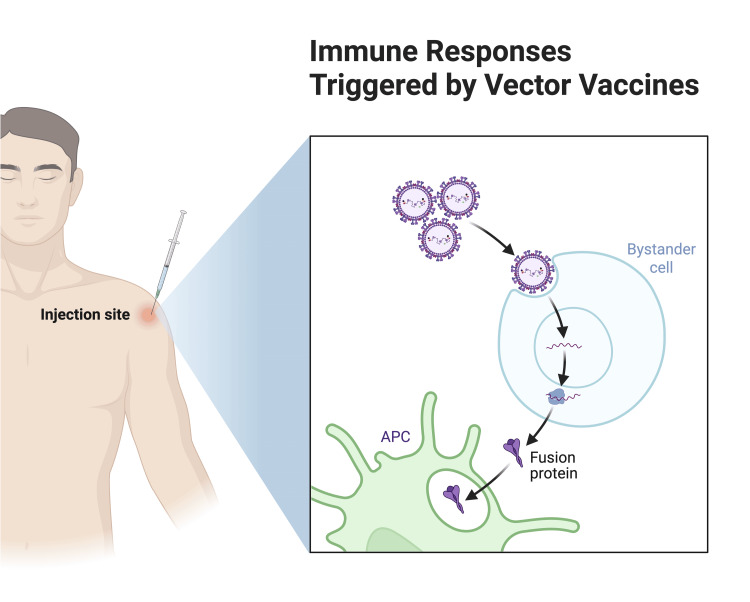
Immune responses triggered by vector-based RSV vaccines RSV: respiratory syncytial virus Image credits: Azhar Hussain, Diana Genis, Wajhat Riaz, Tamara Oz

Viral vector vaccines maintain long-lasting cellular immunity, although their performance can be affected by pre-existing immunity against the chosen vector. These vaccines, such as MVA-BN-RSV, employ a different strategy by inducing powerful CD8+ T-cell responses that lead to durable protection in high-risk populations. They display good tolerability profiles but cause short-term flu-like symptoms, including fever and fatigue, and their effectiveness can decrease due to pre-existing immunity to the viral vector [35]. Studies are ongoing on these vaccines, but they have the potential to generate long-lasting T-cell immunity, which could lead to stronger protection, especially for high-risk groups, including the immunocompromised (Figure 4). A disadvantage of this method is that pre-existing immunity to the viral vector may blunt the immune response [31].

The effectiveness of vaccines varies across age groups due to differences in immune system development and in how they react. In general, RSV infections in infants and newborns lead to severe complications because natural infection fails to provide persistent immunity. Administering Abrysvo to pregnant women in their third trimester significantly lowers the occurrence of medically attended RSV-LRTD in neonates and infants up to six months old, making maternal vaccination an essential method for early protection [30]. In cases where mothers did not receive the RSV vaccine during pregnancy, monoclonal antibody therapies, such as nirsevimab and clesrovimab, protect high-risk infants by neutralizing RSV to provide passive immunity. In addition, RSV vaccines play an essential role for older adults because their weakened immune systems lead to increased hospitalizations and serious disease outcomes. Both GSK’s Arexvy vaccine and Moderna’s mRNA vaccine showed high effectiveness in this group, while Arexvy provided substantial protection against severe RSV cases [33]. Lastly, transplant recipients and individuals with chronic health conditions typically show reduced antibody responses to vaccines, which necessitates additional booster shots or alternate vaccination strategies to maintain an adequate defense [36].

Beyond age-related differences in immune responses, the effectiveness of RSV vaccines also depends on several individual factors, including pre-existing immunity status, overall immune health, and medical conditions. RSV fails to generate long-lasting immunity through natural infection, which makes vaccination essential for enduring protection against the virus [37]. In general, people with weakened immune systems, as well as older adults, require higher-dose vaccines or adjuvanted vaccines to boost their immune responses. Underlying conditions such as chronic obstructive pulmonary disease (COPD), asthma, and cardiovascular disease result in altered immune responses, which make vaccine effectiveness variable among these populations [38].

Regarding safety, RSV vaccines have shown positive results but exhibit varying side effects depending on their delivery method. Patients commonly experience injection-site pain alongside fatigue, headaches, and muscle aches. Guillain-Barré Syndrome (GBS) has been reported as a rare occurrence among older adults who received protein-based RSV vaccines such as Arexvy and Abrysvo [39]. The CDC advises administering Abrysvo to pregnant women during weeks 32 to 36 of pregnancy due to a potential, though not statistically significant, increase in preterm births at less than 32 weeks of gestation and hypertensive disorders [39]. Recent studies show that Beyfortus (nirsevimab) and Enflonsia (clesrovimab-cfor) pose minimal safety risks for infants, but rare hypersensitivity reactions are reported in CDC data from 2024. The CDC and FDA maintain that RSV vaccine benefits exceed its potential risks according to ongoing safety assessments.

The implementation of RSV vaccines requires continuous safety monitoring, given the historical concerns over vaccine-associated enhanced respiratory disease (VAERD) risks observed in early RSV vaccine trials. Stabilized prefusion F-protein formulations decrease risk, but ongoing monitoring remains essential to identify infrequent adverse effects [40]. Surveillance data collected after marketing shows protein-based RSV vaccines caused Guillain-Barré Syndrome in roughly 10 additional cases per million vaccine doses distributed. Two vaccines are particularly associated with increased GBS risk: GSK’s Arexvy and Pfizer’s Abrysvo [39,41].

The FDA requires the addition of warnings regarding the risk of Guillain-Barré syndrome (GBS) to the prescribing information for vaccines Abrysvo and Arexvy following evidence of an increased risk. Research continues to prioritize this issue despite the absence of conclusive proof linking cause and effect. Ongoing surveillance and additional research post-market release are essential for groups such as immunocompromised and frail elderly patients, who were not adequately represented in the main clinical trials, as well as young infants [42]. The CDC and FDA maintain ongoing monitoring of safety records to evaluate possible long-term autoimmune dangers, as well as other RSV vaccination side effects. Pharmacovigilance initiatives provide ongoing monitoring of high-risk groups to demonstrate that vaccination benefits outweigh any potential risks. Future refinement of RSV vaccination strategies and safety optimization for different demographic groups will depend on extensive observational studies and continuous post-licensure surveillance.

Further research is essential to develop universal RSV vaccines that provide protection across diverse populations and strains. Combination vaccines, such as those integrating RSV and influenza prevention, could enhance convenience and coverage. Personalized vaccination strategies should address factors like age, immune status, and comorbidities to protect high-risk groups. Additionally, ensuring global equity in vaccine distribution is critical to prevent disparities in access and health outcomes.

## Conclusions

This review emphasizes the key elements of RSV, including its global impact, limited treatment options, and encouraging developments in vaccine and monoclonal antibody research. RSV poses a significant global health challenge, particularly for infants, the elderly, and immunocompromised individuals. Current treatments are limited, highlighting the urgent need for effective vaccination strategies. Healthcare providers play a crucial role in advocating for RSV vaccination, especially in protecting high-risk populations, to reduce morbidity and mortality. The development of universal RSV vaccines has become a research priority, aiming to deliver broad protection across various populations and strains. Additionally, combination vaccines that integrate RSV and influenza prevention offer promising opportunities to improve coverage and convenience. Personalized vaccination strategies that account for factors such as age, immune status, and comorbidities could further enhance protection for vulnerable groups. Ethical considerations, such as ensuring equitable access to vaccines globally, remain critical in addressing disparities in health outcomes. As technological advancements continue, the future of RSV vaccines is poised to bring transformative solutions, ultimately reducing the burden of RSV and improving public health worldwide.
